# Modeling and Analysis of Single Point Incremental Forming Force with Static Pressure Support and Ultrasonic Vibration

**DOI:** 10.3390/ma12121899

**Published:** 2019-06-13

**Authors:** Lang Bai, Yan Li, Mingshun Yang, Yunbo Lin, Qilong Yuan, Renfeng Zhao

**Affiliations:** School of Mechanical and Precision Instrument Engineering, Xi’an University of Technology, No. 5 South Jinhua Road, Xi’an 710048, Shaanxi, China; bailangdyx@163.com (L.B.); yangmingshun@xaut.edu.cn (M.Y.); yunbolin@126.com (Y.L.); YuanQL@xaut.edu.cn (Q.Y.); zrf20070607@163.com (R.Z.)

**Keywords:** forming force, static pressure support, ultrasonic vibration, analytical modeling, experimental verification

## Abstract

In order to solve the problem of low accuracy caused by instability and springback during the single point incremental forming (SPIF) process, static pressure support (SPS) and ultrasonic vibration (UV) are introduced into the technology for auxiliary forming. In order to qualitatively and quantitatively study the mechanism of static pressure support–ultrasonic vibration-single point incremental forming (SPS-UV-SPIF) force, a typical truncated cone is used as the research object. The working principle and motion rules of the technology are analyzed. The sheet micro-element of the sidewall area is taken as an analysis object. The spatial stress balance equation of the sheet is constructed. The various stresses are integrated and calculated. The forces in each area of the sheet are analyzed and modeled. Finally, an analytical model for SPS-UV-SPIF force is established. The influence law of the static pressure parameter and the vibration parameter on the forming force is obtained. The corresponding SPS system and UV system are designed. The Kistler forming force test system is built. The experimental results are consistent with the theoretical analysis results, which verifies the correctness of the analytical model.

## 1. Introduction

Single point incremental forming (SPIF) is a new technology for sheet metal. The suspended sheet metal is locally dynamically loaded by a specific tool head. The sheet is used to produce an overall cumulative deformation in a dieless and unconstrained state. Finally, the target part is obtained [1]. Since the specific tool head is driven by a CNC (computerized numerical control) machine, this technology can form thin-walled components of various complex shapes. It is flexible, green, fast, and low-cost. However, the partial loading characteristics of the tool head and the suspended clamp characteristics of the sheet make the forming process susceptible to instability and the part susceptible to springback. This reduces forming accuracy, surface quality, and the forming limit of the part [2].

For this reason, researchers in this field have made many attempts to study and improve the forming quality of parts. Shrivastava et al. [3] analyzed the microstructural features in different stages of SPIF through electron back-scattered diffraction (EBSD) and X-ray diffraction (XRD) methods and identified the reasons for typical sheet metal failure. William L. Edwards et al. [4] used experimental heating to process the springback area of the parts. The result showed that heating can effectively reduce the springback of the sheet. Allwood et al. [5] reduced the springback of the part using a pre-manufactured hole in the blank area of the initial sheet metal. Although this method can improve the accuracy of the part, it reduces its rigidity. Parnika Shrivastava et al. [6] preheated the sheet in advance for eliminating the defects in the crystal structure and homogenizing the grain size and distribution of the material. After preheating, the accuracy of the part was clearly improved, and the wall thickness distribution was more uniform. Yanle Li et al. [7] measured the accuracy of the part by the axial error. According to the results, the optimum combination of the process parameters was obtained, which reduced the deformation energy and improved the forming accuracy at the same time. Lu et al. [8] studied the influence of layer spacing on the accuracy of the part. It was found that the smaller the layer spacing, the higher the accuracy of the part. However, the forming time will be significantly increased with smaller layer spacing. A. Fiorentino et al. [9] proposed a method to reduce the geometric error by compensation. This method is an iterative algorithm based on an artificial identification system. Experimental verification was carried out using different tool paths and materials in the non-axisymmetric part. Aqeel Sabree Bedan [10] studied the influence of the ball tool, the hemispherical tool, and the fillet tool on the part accuracy of AL 1050 sheet metal by an NC (Numerical Control) vertical milling machine. It was found that the tool head diameter had the most significant effect on the geometric accuracy. Abolfazl Taherkhani et al. [11] used the group method of data handling (GMDH), an artificial neural network to model the relationships between tool head diameter, layer spacing, sheet thickness, feed speed, axial accuracy, and surface quality. The model was validated by experiments. Italian scholar D. Mundo et al. [12] applied high-frequency vibrations to sheet metal for SPIF. It improved the surface quality and reduced the distortion of parts. Iranian scholar M. Vahdati et al. [13], using experiments, proved the effects of ultrasonic vibration (UV) on SPIF in reducing the axial force, springback, and surface roughness. Iranian scholar S. Amini et al. [14] applied axial high frequency and low amplitude UV to the forming tool to form a 1050 aluminum straight groove. The results showed that UV can reduce the forming force and improve the formability. Japanese scholar Obikawa et al. [15] carried out SPIF experiments of aluminum alloy, steel, and titanium sheets by UV. It was found that UV could significantly improve the geometric accuracy and forming limit of parts. Yanle Li et al. [16] studied the deformation mechanism of sheet metal under UV-SPIF by finite element simulation. The influences of amplitude on the magnitude and distribution of plastic strain were obtained. In addition, some scholars have proposed such technologies as two-point incremental forming [17], high-pressure water jet SPIF [18], electric-assisted SPIF [19], laser-assisted SPIF [20], and electromagnetic incremental forming [21,22] in order to improve the quality of parts by changing the magnitude and action of forming force.

Scholars have done research in controlling forming accuracy and improving forming quality and have attained certain achievements. In order to further improve forming quality, in this paper, UV was applied to the tool head to improve the residual stress distribution, thereby reducing the springback of the part. The static pressure support (SPS) was also applied to the retaining sheet to change the suspension state, thereby improving the axial accuracy of the part. For this reason, the technology of simultaneously introducing ultrasonic vibration and static pressure support into the SPIF is proposed for the first time. Under the action of two auxiliary technologies, the magnitude and action of the forming force have changed. In order to study the SPS–UV single-point incremental forming (SPS-UV-SPIF) force, the working principle and motion rules of the technology are studied. The sheet micro-element of the formed contact area was taken as the analysis object. The spatial stress balance equation of the sheet micro-element was constructed. The stresses of the various directions were integrated, and the forming forces of the contact area were obtained and expressed analytically. An analytical model of the forming force of SPS-UV-SPIF was constructed, and the influence law of static pressure parameters and vibration parameters on the forming force was obtained. Finally, it was verified by experiments.

**Figure 1:**
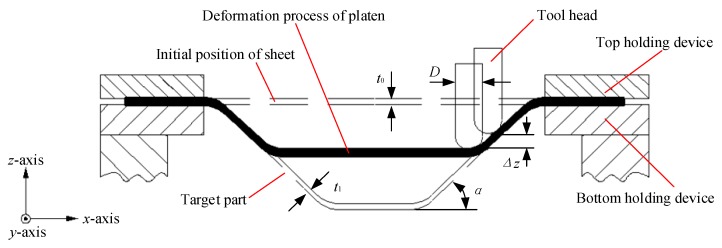
The technology principle of single-point incremental forming (SPIF).

The forming principle of SPS-UV-SPIF technology is based on SPIF. To study the SPS-UV-SPIF, it is necessary to first understand the forming principle of the SPIF, as shown in Figure 1. The sheet to be formed is suspended and held on the table of the machine tool by the top holding device and bottom holding device. The initial thickness of the sheet is *t*_0_. The thickness of the target part after cumulative forming by the tool head of diameter *D* is *t*_1_. The layer spacing of layer-by-layer cumulative forming is *Δz*. The forming angle of the target part is *α*.

**Figure 2:**
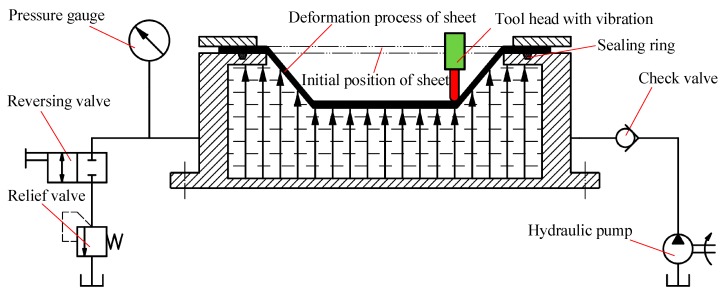
The technology principle of static pressure support–ultrasonic vibration single-point incremental forming (SPS-UV-SPIF).

The forming principle of SPS-UV-SPIF technology is shown in Figure 2. Based on the SPIF technology, the longitudinal UV is applied to the tool head. The back of the sheet is supported by the static pressure. Ultrasonic vibration is realized by an ultrasonic generator, a transducer, and an amplitude transformer. After the sheet material is subjected to the UV energy, the deformation ability and residual stress changes. This helps to improve the accuracy error caused by the springback of the part. The SPS is realized by a sealing ring, a relief valve, a hydraulic valve, a pressure gauge, a tank, and a check valve. The hydraulic oil in the tank is delivered to the seal holders by the hydraulic pump. The hydraulic oil under the action of the relief valve keeps the back support pressure of the entire forming process constant, which is equivalent to providing a flexible support for the forming sheet, as shown by the black arrows on the back of the sheet. This helps to improve the accuracy error caused by the suspended sheet.

## 2. Methods

Based on the relationship model between forming force and vibration parameters [23], the micro-element method is used as the forming force modeling method of SPS-UV-SPIF technology. The idea of this method is from micro to macro, partial to whole. It can discretely simplify complex problems. The modeling process is performed in three steps. The first is to establish a balance equation for the forming force. The second is to establish the geometric equation of the motion mechanism. The third is to combine the first two equations to obtain the mechanical analysis model between the forming force and the forming parameters, including static pressure parameters and vibration parameters.

### 2.1. Mechanical Balance Equation

**Figure 3:**
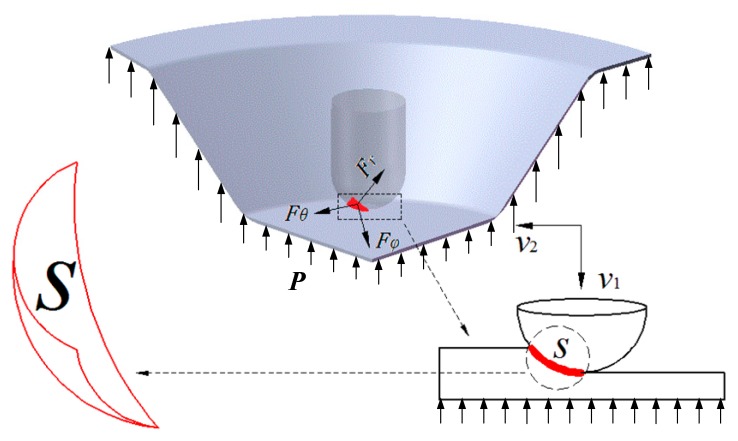
The contact area of the sheet and tool head [23].

Figure 3 is a schematic figure of the contact area *S* between the tool head and the sheet. The sheet in the figure is a quarter-cone section. It is subjected to the vibrational impact force from the upper tool head and the SPS force from the lower hydraulic pressure. The sheet becomes a curved sector contact area under the action of the normal force *F_r_*, the circumferential force *F_θ_*, the radial force *F_φ_*, and the supporting force *P*. The tool head impacts the sheet at a speed *v*_1_ while having a feed speed *v*_2_ in the direction along the forming track.

**Figure 4:**
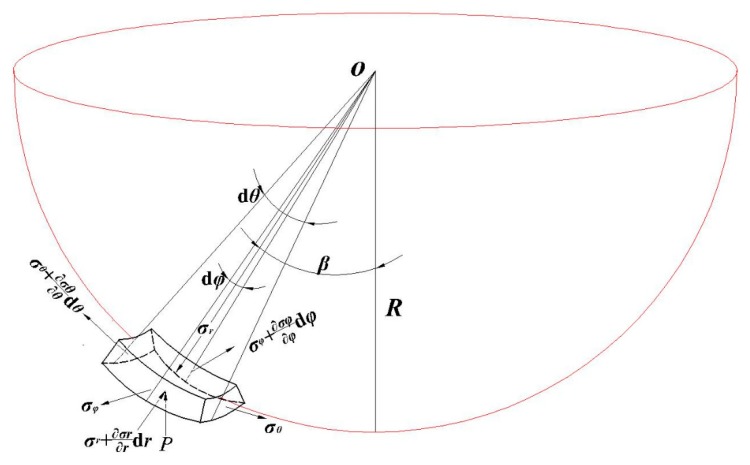
Stress components of the sheet micro-element in spherical coordinates.

The micro-element of the contact area was taken as the research object. A spherical coordinate system was constructed to analyze the balance relationship of stress in each direction, as shown in Figure 4. *σ_r_*, *σ_θ_*, and *σ_φ_* are the normal stress, the circumferential stress, and the radial stress of the micro-element, respectively. d*θ* and d*φ* are the angles of the opposite sides in the height direction of the micro-element, respectively. The *β* is the declination of the micro-element center relative to the tool head. *R* is the tool head radius. The *t* is the thickness of the micro-element sheet. The stress balance equation of the spherical coordinate system was established vertically in the micro-element, as in Equation (1).

(1) $$\begin{array}{l} -\sigma_{r}\cdot \left(R\cdot d\theta \right)\cdot \left(R\cdot d\varphi \right)+\left(\sigma_{r}+\frac{\partial \sigma_{r}}{\partial r}dr\right)\cdot {\left(R+t\right)}^{2}\cdot d\theta \cdot d\varphi +\sigma_{\varphi }\cdot \sin\frac{d\varphi }{2}\cdot \left(\pi \cdot t^{2}\cdot \frac{d\theta }{2\pi }+2\pi \cdot t\cdot R\cdot \frac{d\theta }{2\pi }\right)\\ +\left(\sigma_{\varphi }+\frac{\partial \sigma_{\varphi }}{\partial \varphi }d\varphi \right)\cdot \sin\frac{d\varphi }{2}\cdot \left(\pi \cdot t^{2}\cdot \frac{d\theta }{2\pi }+2\pi \cdot t\cdot R\cdot \frac{d\theta }{2\pi }\right)+\sigma_{\theta }\cdot \sin\frac{d\theta }{2}\cdot \left(\pi \cdot t^{2}\cdot \frac{d\varphi }{2\pi }+2\pi \cdot t\cdot R\cdot \frac{d\varphi }{2\pi }\right)\\ +\left(\sigma_{\theta }+\frac{\partial \sigma_{\theta }}{\partial \theta }d\theta \right)\cdot \sin\frac{d\theta }{2}\cdot \left(\pi \cdot t^{2}\cdot \frac{d\varphi }{2\pi }+2\pi \cdot t\cdot R\cdot \frac{d\varphi }{2\pi }\right)+{\left(R+t\right)}^{2}\cdot d\theta \cdot d\varphi \cdot P\cdot \cos\beta =0 \end{array}$$

The third-order micro term is eliminated, so the following equation can be obtained:

(2) $$\sigma_{r}\cdot d\theta \cdot d\varphi +\sigma_{\varphi }\cdot \sin\frac{d\varphi }{2}\cdot d\theta +\sigma_{\theta }\cdot \sin\frac{d\theta }{2}\cdot d\varphi +{\left(R+t\right)}^{2}\cdot d\theta \cdot d\varphi \cdot P\cdot \cos\beta =0.$$

The above balance equation applies to any micro-element in the contact area. In the strain study of the truncated cone part, it was found that the actual deformation of the sheet in the circumferential direction was much smaller than that of the radial stretching and the vertical thinning [23]. Therefore, the circumferential stress *σ_θ_* is assumed to be zero. That is, Equation (2) can be simplified to

(3) $$\sigma_{r}\cdot \varphi +\sigma_{\varphi }\cdot \sin\frac{\varphi }{2}+{\left(R+t\right)}^{2}\cdot \varphi \cdot P\cdot \cos\beta =0.$$

According to the yield criterion of the Mises plane deformation, the following relation is known:

(4) $$\sigma_{r}-\sigma_{\varphi }=k\cdot \sigma_{s}=\frac{2}{\sqrt{3}}\sigma_{s}.$$

Equations (3) and (4) are solved simultaneously to obtain the values of *σ_φ_* and *σ_r_*, as shown in the following equations:

(5) $$\left\{ \begin{array}{l} \sigma_{\varphi }=\frac{{\left(R+t\right)}^{2}\cdot \varphi \cdot P\cdot \cos\beta +\frac{2}{\sqrt{3}}\sigma_{s}\cdot \varphi }{\varphi +\sin\frac{\varphi }{2}}\\ \sigma_{r}=\frac{{\left(R+t\right)}^{2}\cdot \varphi \cdot P\cdot \cos\beta +\frac{4}{\sqrt{3}}\sigma_{s}\cdot \varphi +\frac{2}{\sqrt{3}}\sigma_{s}\cdot \sin\frac{\varphi }{2}}{\varphi +\sin\frac{\varphi }{2}} \end{array}\right..$$

### 2.2. The Geometric Equation of the Contact Area

**Figure 5:**
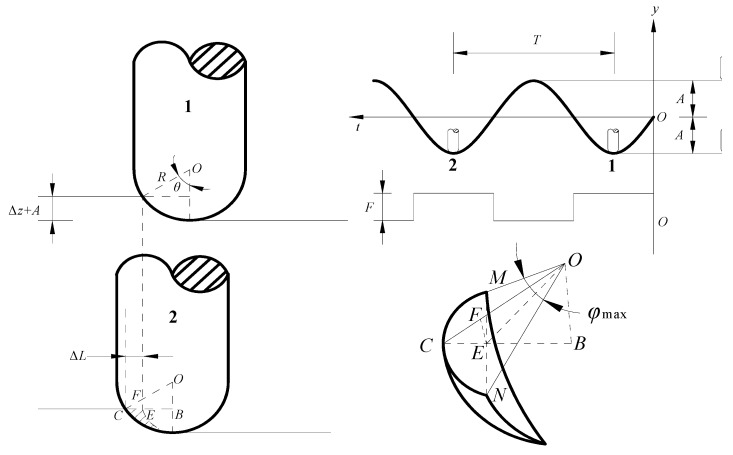
Geometric diagram of the tool head movement.

It is necessary to study the maximum values of ∠*β* and ∠*φ* in Equation (5) to obtain the normal force *F_r_* and the radial force *F_φ_*. Therefore, the motion rule of the tool head when forming the sheet material was analyzed. The ∠*β* and ∠*φ* were obtained according to the geometric relationship. Figure 5 is the geometric relationship of the contact area. The T is the tool head vibration period. The *A* is the vibration amplitude. The vibration trajectory is sinusoidal. The *Δz* is the layer spacing. The tool head has the largest amplitude at time 1 and time 2. At this moment, the forming force *F* also reaches the maximum, that is, the resultant force in the three directions reaches the maximum. Since one vibration period *f* is very short, it is considered that *F* is a constant force.

Time 1 and time 2 indicate the start and end time points of a cycle, and the horizontal displacement of the tool head in this cycle is *ΔL*, as shown in Equation (6).

(6) $$\Delta L=CE=\frac{v_{2}}{f}.$$

The maximum values of ∠*β* and ∠*φ* can be determined according to the geometric relationship in Figure 5. The values of *β*_max_ and *φ*_max_ can be calculated from Equations (7) and (8) as follows:

(7) $$\beta_{max}=\arccos\frac{OB}{OC}=\frac{R-\Delta z-A}{R}$$

(8) $$\varphi_{max}=2\arccos\frac{OE}{R}=2\arccos\frac{\sqrt{R^{2}+\frac{v_{2}{}^{2}}{f^{2}}\cdot \frac{1}{3600}-\frac{v_{2}}{f}\cdot \frac{1}{30}\sqrt{\left(\Delta z+A\right)\cdot \left(2R-\Delta z-A\right)}}}{R}.$$

### 2.3. Forming Force Analytical Model and Coordinate Transformation

The nature of the radial force *F_φ_* and the normal force *F_r_* is the integral of *σ_φ_* and *σ_r_* on the contact surface. The contact surface is related to ∠*β* and ∠*φ*. The constant intervals of ∠*β* and ∠*φ* are [0, *β*_max_] and [0, *φ*_max_], respectively. The radial force *F_φ_* and the normal force *F_r_* can be obtained as follows:

(9) $$F_{\varphi }=\int_{0}^{\beta_{{}_{\max}}}\int_{0}^{\varphi_{\max}}\frac{{\left(R+t\right)}^{2}\cdot \varphi \cdot P\cdot \cos\beta +\frac{2}{\sqrt{3}}\sigma_{s}\cdot \varphi }{\varphi +\sin\frac{\varphi }{2}}d\beta d\varphi$$

(10) $$F_{r}==\int_{0}^{\beta_{{}_{\max}}}\int_{0}^{\varphi_{\max}}\frac{{\left(R+t\right)}^{2}\cdot \varphi \cdot P\cdot \cos\beta +\frac{4}{\sqrt{3}}\sigma_{s}\cdot \varphi +\frac{2}{\sqrt{3}}\sigma_{s}\cdot \sin\frac{\varphi }{2}}{\varphi +\sin\frac{\varphi }{2}}d\beta d\varphi .$$

**Figure 6:**
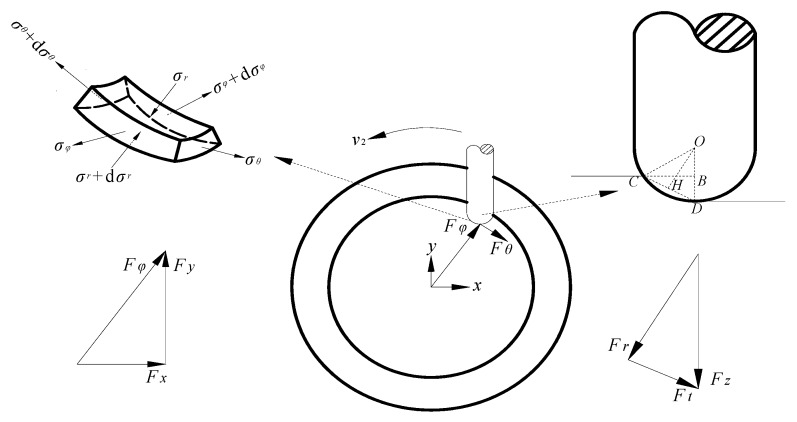
Relationship of the forming forces in different coordinate systems.

In this paper, the correctness of the analytical model is verified experimentally. However, the coordinate system of the experimental measuring force is inconsistent with the analytical modeling coordinate system, so the two cannot be directly used for comparison verification. The relationship between the two coordinate systems needs to be analyzed. Their interrelationships were established to construct a mathematical relationship between analytical force and experimental force for the purpose of comparative verification. The experimental coordinate system is based on the space rectangular coordinate system of the *x–y* plane and the vertical *z*-axis of the machine table. The analytical modeling coordinate system is based on the spherical coordinate system of the sheet micro-element in the contact area. The relationship between the two spatial geometries was constructed, as shown in Figure 6.

In the process of analytical modeling, the *F_θ_* was idealized to zero. The sheet was then only subjected to the radial force *F_φ_* in the *x–y* plane. Therefore, *F_φ_* is the resultant force of *F_x_* and *F_y_*, as shown in Equation (11). The tool head is taken as the research object in Figure 6. The auxiliary line *OH* is perpendicular to the auxiliary line *CD*, and the foot is *H*. The relational Equation (12) was obtained from the geometric relationship, so the relationship Equation (13) between the normal force *F_r_* and the axial force *F_z_* was obtained. Since the analytical model was an ideal model that did not consider friction, the downward frictional force *F_t_* along the contact area *S* was not considered

(11) $$F_{\varphi }=\sqrt{F_{x}{}^{2}+F_{y}{}^{2}}$$

(12) $$\frac{F_{r}}{F_{z}}=\frac{OH}{OD}=\frac{CB}{CD}=\sqrt{1-\frac{\Delta z+A}{2R}}$$

(13) $$F_{r}=F_{z}\cdot \sqrt{1-\frac{\Delta z+A}{2R}}.$$

## 3. Experiments

In order to verify the analytical model, the corresponding experiment was designed. SPS systems and UV systems were developed to verify the variation of the forming force with respect to static pressure parameters and vibration parameters.

### 3.1. Experimental Equipment and Materials

A three-axis vertical CNC milling machine was selected as the experimental platform. The forming tool was a hemispherical tool head made of X210CrW12 tungsten high speed steel. Its high rigidity, hardness, and wear resistance can effectively ensure the surface quality of the part and avoid the interference of tool head deformation on the accuracy of the part. AL1060 aluminum alloy was selected as the forming sheet with a size of *φ*135 mm, and the material parameters are shown in Table 1. The lubricant was a Fuchs Renoform Fw50s metal forming lubricant suitable for aluminum alloy stretching. The target part had a truncated cone depth of 24 mm. A Kistler 9257B three-direction dynamometer was used as the forming force detecting device. The sensor was responsible for collecting the three-direction force, and the force signal was amplified by the charge amplifier and transmitted to the data acquisition instrument. Finally, the display analysis of the three-direction force was realized by Vib’SYS software.

### 3.2. Static Pressure Support System

**Figure 7:**
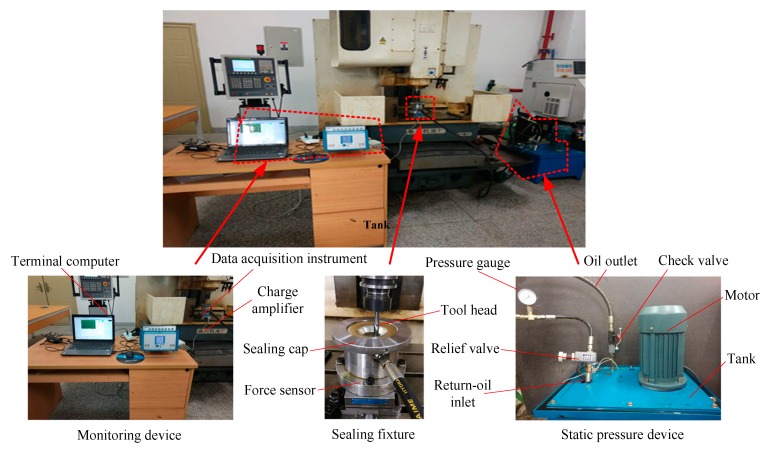
Static pressure support (SPS) system.

The introduction of SPS in the SPIF technology provided a constant support for the back of the sheet, solving the axial geometric error caused by the suspended clamp. The SPS system is shown in Figure 7, and the static pressure oil is L-HM46. The monitoring device can monitor the stability of the forming process while collecting the forming force, and can ensure the controllability of the experimental process. The sealing fixture is fixed on the machine table and is responsible for the clamp of the sheet and the sealing of the static pressure oil. The motor provides a continuously stable static pressure oil to the seal fixture. The relief valve is the key to achieving constant pressure in the sealing fixture. It can return excess static pressure oil to the tank under the set static pressure condition, keeping the circulation of static pressure oil in the sealing fixture.

### 3.3. Ultrasonic Vibration System

**Figure 8:**
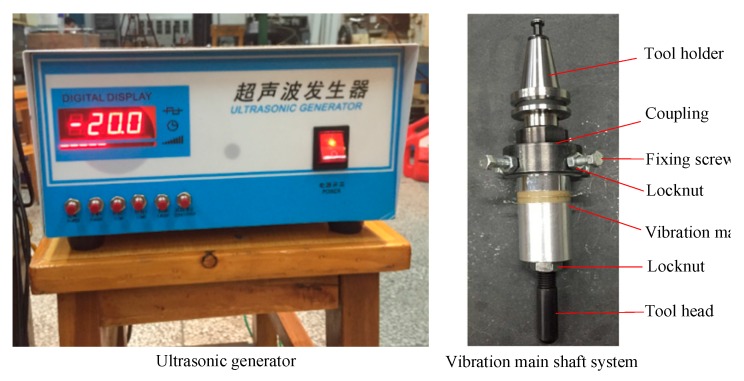
Ultrasonic vibration system.

Ultrasonic vibration (UV) technology was also introduced in the SPIF. The UV was used to improve the deformability and residual stress distribution of the sheet material to solve the geometric accuracy error caused by the springback of the part. The UV system is mainly composed of a vibration main shaft system and an ultrasonic generator, as shown in Figure 8. The vibration main shaft consisted of a transducer, an amplitude transformer, and a main shaft shell. The transducer and amplitude transformer were mounted as internal components in the main shaft shell. One end of the vibration main shaft was connected to the tool holder, and the other end was connected to the tool head. The connection to the tool holder was required by a coupling that was a stepped sleeve. The upper end and the tool holder were coaxially connected by a connecting thread. The lower end was connected with the set screws in four directions, and the locknut to ensure the coaxiality of the connection between the tool holder and the vibration main shaft. The stepless frequency modulation ultrasonic generator was selected as the vibration generator to quantitatively study the influence of frequency and amplitude on the forming force. The frequency can be continuously adjusted from 13 to 75 kHz with an adjustment precision of 0.1 kHz. The generator was connected to the vibration main shaft via wires.

## 4. Results and Discussion

It can be seen from the analytical model that the forming parameters affecting the radial force *F_φ_* and the normal force *F_r_* include the static pressure *P*, the amplitude *A*, the frequency *f*, the tool head radius *R*, the layer spacing *Δz*, the sheet thickness *t*, and the feed speed *v*_2_. In order to study the influence of various parameters on the forming force of SPS-UV-SPIF, the control variable method was used to compare the analytical results and experimental results. Thereby, the mechanism of the influence of each parameter on the forming force was analyzed and discussed.

**Figure 9:**
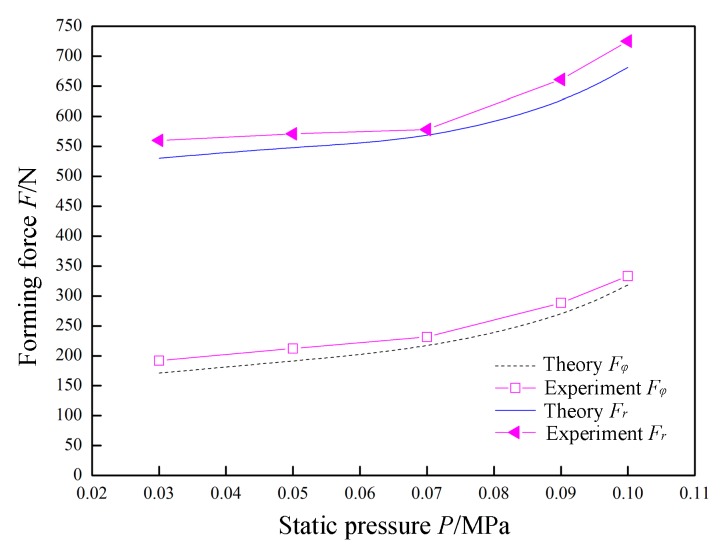
The effect of static pressure on forming force.

**Figure 10:**
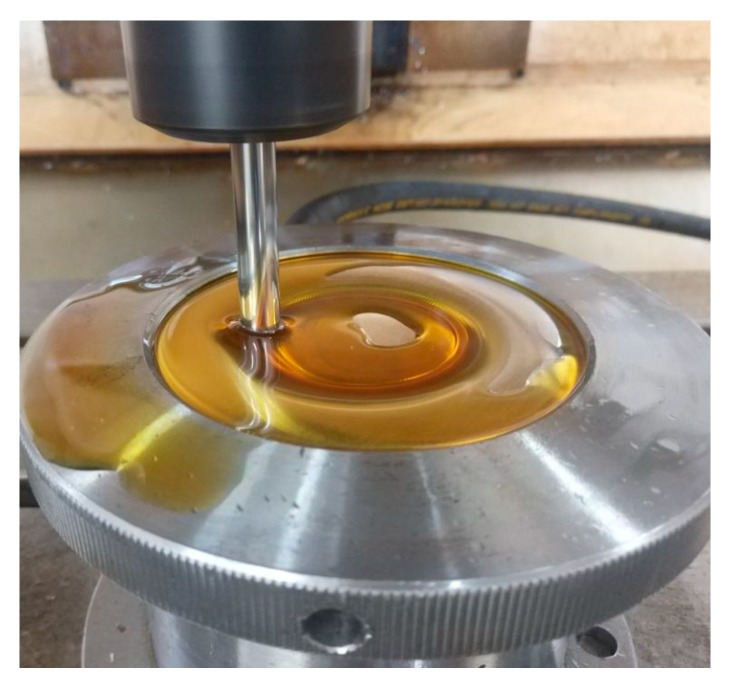
Significant upward pre-deformation.

In Figure 9, *A =* 0.03 mm, *f =* 30 kHz, *R =* 5 mm, *Δz =* 1.0 mm, *t =* 1 mm, and *v*_2_ = 200 mm/min were constant, and the static pressure *P* was variable. The variation laws of the forming forces with respect to the static pressure *P* are obtained in theory and through experiments. The two trends are basically the same, and the relationship between static pressure *P*, radial force *F_φ_*, and normal force *F_r_* in the analytical model is verified. As the static pressure *P* increases, the supporting force on the back of the sheet becomes increasingly large, such that the radial force *F_φ_* and normal force *F_r_* increase with the increase of the static pressure *P*. When the static pressure exceeds 0.07 MPa, excessive static pressure causes the sheet to undergo an upward pre-deformation. As a result, the tool head needs to overcome more deformation energy when forming the sheet, so the increase of the forming force becomes large. When the static pressure exceeds 0.11 MPa, the sheet will exhibit a very significant upward pre-deformation during the initial forming stage, which is disadvantageous for forming, as shown in Figure 10. Therefore, the upper limit of the static pressure parameter is set to 0.1 MPa.

**Figure 11:**
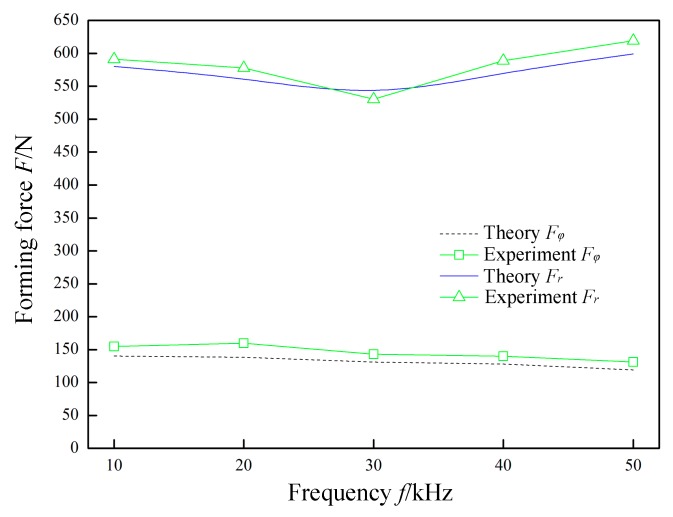
The effect of frequency on forming force.

In Figure 11, *P* = 0.07 MPa, *A* = 0.03 mm, *R* = 5 mm, *Δz* = 1.0 mm, *t* = 0.8 mm, and *v*_2_ = 200 mm/min were constant, and the frequency *f* was variable. The variation laws of the forming forces with respect to the frequency *f* are obtained in theory and through experiments. The normal force *F_r_* shows a nonlinear change with an increase in the frequency *f*. This is because, when the frequency *f* is lower than 30 kHz, as the frequency *f* increases, the sheet is subjected to an increase in the impact density of the tool head. The material absorbs more ultrasonic energy, which in turn changes the rheological mechanism and structure evolution mechanism of the material. Macroscopically, the softening of the material and the forming limit are improved. At this time, the normal force *F_r_* decreases as the frequency *f* increases. When the frequency *f* is greater than 30 kHz, the instantaneous deformation of the sheet increases. More material participates in the deformation, which causes the corresponding normal force *F_r_* to increase as the frequency *f* increases. However, the radial force *F_φ_* linearly decreases as the frequency *f* increases. This is because, as the frequency *f* increases, the single impact deformation amount also decreases linearly.

**Figure 12:**
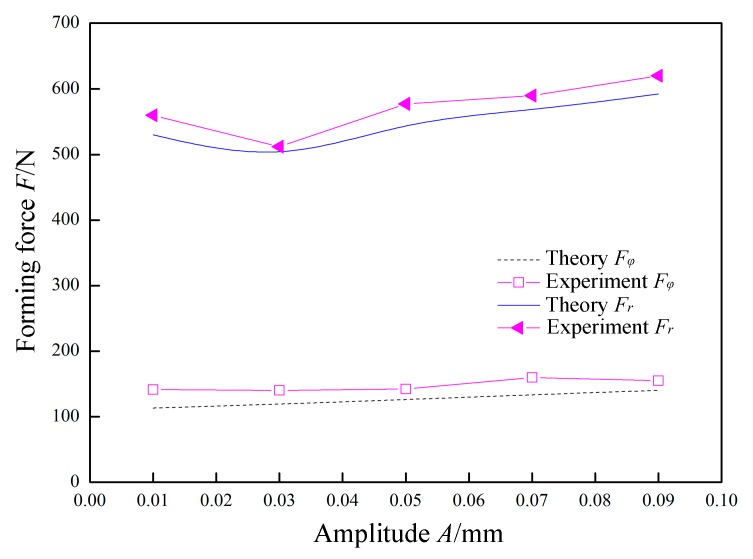
The effect of amplitude on forming force.

In Figure 12, *P* = 0.07 MPa, *f* = 30 kHz, *R* = 5 mm, *Δz* = 1.0 mm, *t* = 0.8 mm, and *v*_2_ = 200 mm/min were constant, and the amplitude *A* was variable. The variation laws of the forming forces with respect to the amplitude *A* are obtained in theory and experiment. The normal force *F_r_* exhibits a nonlinear change with an increase in the amplitude *A*. This is because, when the amplitude *A* is lower than 0.03 mm, the impact of the tool head on the sheet is low. The yield limit of the material is not reached. At the moment when the tool head is separated from the sheet material, the upward springback of the sheet material causes the tool head to continuously contact the sheet, and the normal force *F_r_* is decreased. When the amplitude *A* is around 0.03 mm, the impact of the tool head just exceeds the yield limit of the sheet material. The tool head is not subjected to continuous contact by the springback of the sheet when it is separated from the sheet. When the amplitude *A* is larger than 0.03 mm, as the amplitude increases, the amount of deformation in the thickness direction of the sheet increases, and the corresponding normal force *F_r_* also increases. However, the radial force *F_φ_* increases linearly with the increase of the amplitude A. This is because the increase in amplitude accordingly causes the amount of radial deformation to linearly increase.

**Figure 13:**
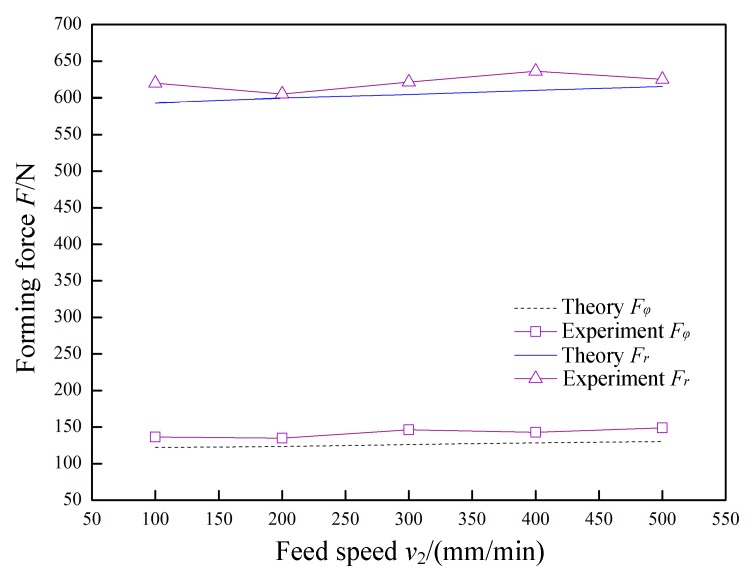
The effect of feed speed on forming force.

In Figure 13, *P* = 0.07 MPa, *A* = 0.03 mm, *f* = 30 kHz, *R* = 5 mm, *Δz* = 1.0 mm, and *t* = 0.8 mm were constant, and the feed speed *v*_2_ was variable. The variation laws of the forming forces with respect to the feed speed *v*_2_ are obtained in theory and experiment. The normal force *F_r_* and the radial force *F_φ_* linearly increase as the feed speed *v*_2_ increases, but the increase amplitude is low. Because the time required for the tool head to impact the sheet is very short, the change in feed speed *v*_2_ does not substantially affect the amount of deformation of the sheet material by a single impact. Therefore, within the allowable range of actual forming, the feed speed *v*_2_ should be increased as much as possible to improve the forming efficiency.

**Figure 14:**
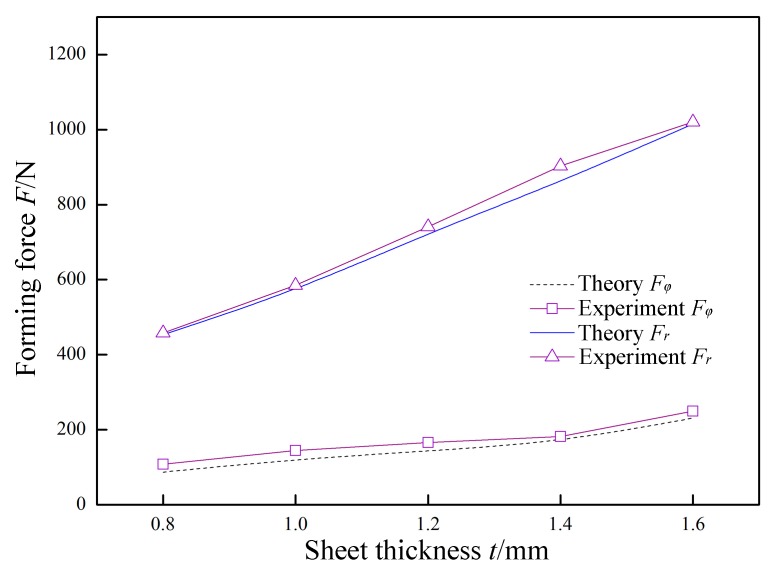
The effect of sheet thickness on forming force.

In Figure 14, *P* = 0.07 MPa, *A* = 0.03 mm, *f* = 30 kHz, *R* = 5 mm, *Δz* = 1.0 mm, and *v*_2_ = 200 mm/min were constant, and the sheet thickness *t* was variable. The variation laws of the forming forces with respect to the sheet thickness *t* are obtained in theory and experiment. The normal force *F_r_* and the radial force *F_φ_* linearly increase as the thickness *t* increases, and the increase amplitude is large. Since the other parameters are unchanged, increasing the sheet thickness *t* is equivalent to increasing the deformation resistance. That is, the forming force is increased.

**Figure 15:**
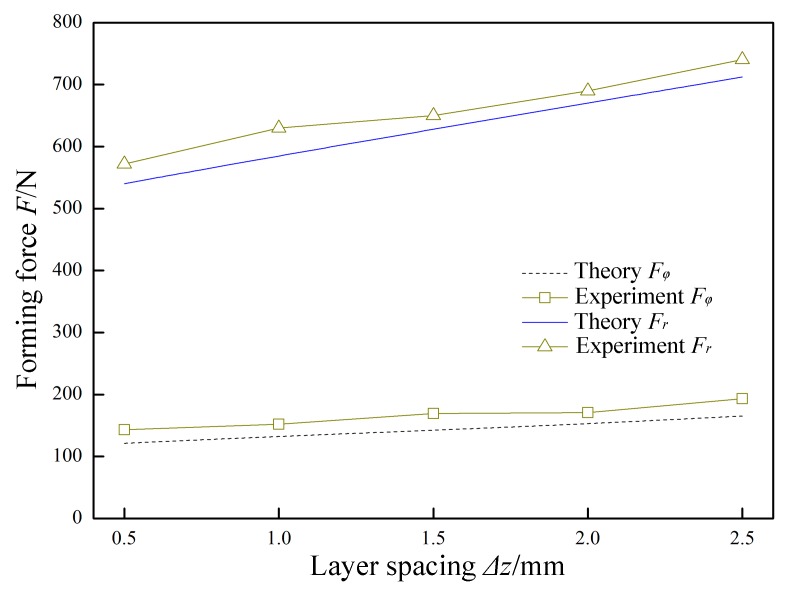
The effect of layer spacing on forming force.

In Figure 15, *P* = 0.07 MPa, *A* = 0.03 mm, *f* = 30 kHz, *R* = 5 mm, *t* = 0.8 mm, and *v*_2_ = 200 mm/min were constant, and the layer spacing *Δz* was variable. The variation laws of the forming forces with respect to the layer spacing *Δz* are obtained in theory and experiment. Both the normal force *F*_r_ and the radial force *F*_φ_ linearly increase as the layer spacing *Δz* increases. This is because increasing the layer spacing *Δz* causes a larger amount of sheet deformation. The increase amplitude is less than the increase amplitude caused by the sheet thickness *t*, and is higher than the increase amplitude caused by the feed speed *v*_2_. The excessive *Δz* will cause bending and springback of the part, and a *Δz* value that is too low will reduce the forming efficiency. Therefore, the use of a suitable *Δz* at different characteristics can improve the forming efficiency while maintaining the quality of the part.

**Figure 16:**
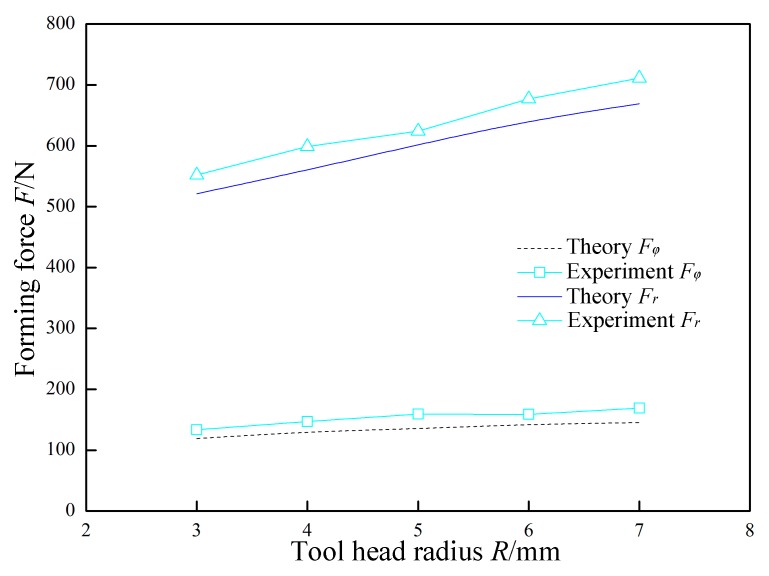
The effect of tool head radius on forming force.

In Figure 16, *P* = 0.07 MPa, *A* = 0.03 mm, *f* = 30 kHz, *Δz* = 1.0 mm, *t* = 0.8 mm, and *v*_2_ = 200 mm/min were constant, and the tool head radius *R* was variable. The variation laws of the forming forces with respect to the tool head radius *R* are obtained in theory and experiment. Both the normal force *F*_r_ and the radial force *F*_φ_ linearly increase as the tool head radius *R* increases. This is because increasing the tool head radius *R* increases the amount of deformation of the sheet. In the forming process, an *R* value that is too high is not conducive to the formation of complex parts, and an *R* value that is too low is likely to cause stress concentration. Therefore, the dimension of the tool head should be selected according to the actual forming characteristics.

## 5. Conclusions

This paper proposes a new type of SPS–UV SPIF technology that can improve the accuracy of parts. In order to study the technology in depth, the mechanical analysis model was established and analyzed based on the forming principle and the motion rule. Finally, the analytical model was verified by the corresponding experiments, and the following conclusions were obtained:

(1) According to the analytical model, it can be seen that the normal force *F_r_* and the radial force *F_φ_* are related to the static pressure *P*, the amplitude *A*, the frequency *f*, the tool head radius *R*, the layer spacing *Δz*, the sheet thickness *t*, and the feed speed *v*_2_. There is also a difference in their degree of relevance.

(2) According to the comparison between the experimental results and the theoretical results, it was found that the actual variation trend of the normal force *F_r_* and the radial force *F_φ_* with respect to each parameter tends to be consistent with the theoretical results, which confirms the correctness of the analytical model.

(3) According to the analysis results, the effects of static pressure parameters and vibration parameters on the forming force are nonlinear. As the static pressure increases, the normal force *F_r_* does not continue to increase linearly. When the static pressure exceeds 0.07 MPa, the sheet will have an upward pre-deformation, resulting in a sudden increase in the forming force. When the amplitude *A* is around 0.03 mm, the impact force of the tool head just exceeds the yield limit of the sheet material and the forming force at this time is the lowest. When the frequency *f* is around 30 kHz, the softening effect of the material is best and the forming force at this time is the lowest.

## Figures and Tables

**Table 1:** The main chemical composition of the AL1060 aluminum alloy (%, mass fraction). [23].

Chemical Element	Al	Cu	Ti	Zn	Si	Mg	Mn	Fe
Content (%)	99.63	0.03	0.02	0.03	0.12	0.02	0.01	0.14

## Abbreviations

SPIF	Single Point Incremental Forming
UV	Ultrasonic Vibration
SPS	Static Pressure Support
SPS-UV-SPIF	Static Pressure Support–Ultrasonic Vibration-Single Point Incremental Forming
CNC	Computerized Numerical Control
NC	Numerical Control
*t*_0_	Initial Thickness of the Sheet
*t*_1_	Thickness of the Target Part
*D*	Diameter of Tool Head
*Δz*	Layer Spacing
*α*	Forming Angle of the Target Part
*S*	The Contact Area
*F_r_*	Normal Force
*F_θ_*	Circumferential Force
*F_φ_*	Radial Force
*P*	Static Pressure Support Force
*v*_1_	Impact Speed of Tool Head
*v*_2_	Feed Speed of Tool Head
*σ_r_*	Normal Stress
*σ_θ_*	Circumferential Stress
*σ_φ_*	Radial Stress
d*θ*	Angle between the Sides of the Micro-Element
d*φ*	Another Angle between the Sides of the Micro-Element
*β*	Declination of the Micro-Element Center Relative to the Tool Head
*R*	Tool Head Radius
*t*	Thickness of the Micro-Element
*F*	Forming Force
*T*	Vibration Period
*ΔL*	Horizontal Displacement of the Tool Head in One Vibration Period
*β*_max_	Maximum Values of ∠*β*
*φ*_max_	Maximum Values of∠*φ*
*F_x_*	Horizontal Force of the *x-y* Plane
*F_y_*	Horizontal Force of the *x-y* Plane
*F_z_*	Axial Force
*F_t_*	Frictional Force
